# Split anterolateral thigh free flaps for bilateral open fracture ankle defects: A one donor, two flap solution

**DOI:** 10.1016/j.jpra.2025.08.029

**Published:** 2025-08-23

**Authors:** Elizabeth Wilson, Calver Pang, Charles Yuen Yung Loh

**Affiliations:** a Department of Plastic and Reconstructive Surgery, Addenbrookes Hospital, Cambridge University Hospitals, Hills Rd, Cambridge CB2 0QQ, United Kingdom; b Department of Surgical Biotechnology, Division of Surgery and Interventional Science, Faculty of Medical Sciences, University College London, Gower St, London WC1E 6BT, United Kingdom

**Keywords:** Lower limb trauma, Lower limb reconstruction, Ankle defects, Split alt flap, ALT flap

## Abstract

Soft tissue reconstruction in bilateral ankle defects associated with lower limb trauma is complex and challenging. There is limited application of split free anterolateral (ALT) thigh flaps in lower limb reconstruction. This case report highlights a novel and efficient method that utilised a single donor site for the harvest of split ALT, with independent pedicles, to reconstruct bilateral simultaneous medial and lateral ankle defects.

## Introduction

Bilateral soft tissue defects of the ankle secondary to open fractures and traumatic injury pose a particularly complex reconstructive challenge, especially when internal fixation is required on both the medial and lateral aspects, necessitating reliable soft tissue coverage. Local flap options are very limited in the distal third of the leg, especially for coverage of extensive soft tissue defects. We present a novel and efficient approach that utilises a single donor site for the harvest of two free flaps, termed the split anterolateral (ALT) flap. The ALT flap is uniquely suited for this purpose due to its multiple perforators from various origins. Despite anatomical variation, a well-designed split skin paddle can facilitate the harvest of two distinct flaps. Perforators originating from either the descending branch or oblique branch of the lateral circumflex femoral artery (LCFA) may be incorporated into the design. The descending branch itself may be divided, with each segment supplying a separate skin paddle. Alternatively, the transverse branch of the descending circumflex artery or the tensor fascia lata branch may be used to supply the overlying skin paddle. This case illustrates the feasibility and versatility of the split ALT in addressing complex bilateral ankle defects.

## Case presentation

A 50-year-old male was involved in an incident at work and sustained a crush injury to his left ankle from a metal pipe. This resulted in a Gustillo-Anderson grade IIIa open pilon tibia fracture of his ankle with a seven-centimetre laceration medially and puncture wounds on the antero-lateral aspect. Prior to his injury he was fit and well, with hypertension controlled on ramipril (Altace) and no allergies. He smoked 20 cigarettes per day.

During initial debridement, the lateral wound was noted to expose the underlying fibula fracture, while the medial aspect demonstrated distal degloving with contused overlying skin. Given the extent and complexity of the injuries, the patient was deemed a candidate for open reduction and internal fixation of the pilon fracture, in conjunction with soft tissue reconstruction using free flap coverage. Owing to the presence of the two anatomically distinct defects, a split ALT was planned to achieve simultaneous bilateral coverage from a single donor site.

**Figure 1:**
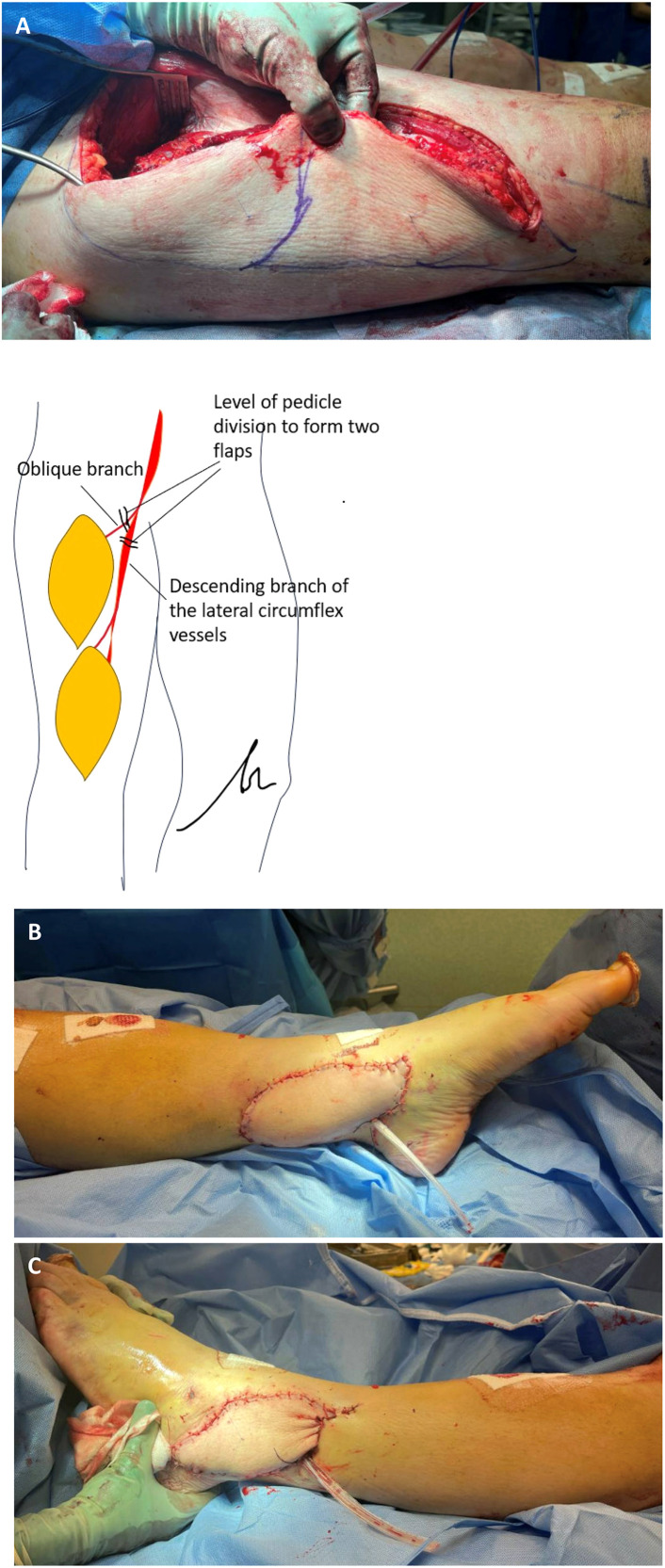
A – Design of split ALT free flap. B – Medial ankle flap. C – Lateral ankle flap.

The patient underwent a second-stage procedure where the orthopaedic team performed fracture fixation using a plate to stabilise the distal articular fragment, tibial nail, a lateral locking plate and a separate plate for syndesmosis stabilisation. A fasciocutaneous ALT flap was raised from the contralateral thigh and divided into two separate skin paddles, each supplied by distinct vascular pedicles (Fig. 1). A CT angiogram of the lower limb was performed prior to surgery for assessment of recipient vessels and a handheld doppler assessment of perforators during flap design. The lateral ankle flap was designed with two perforators from the descending branch of the LCFA, while the medial ankle flap incorporated two perforators arising from the oblique branch. Although both branches were proximally connected, the two flaps were harvested as separate pedicles prior to the confluence of the branches, thereby enabling the creating of two distinct pedicles for separate anastomoses. Microvascular anastomoses were performed with the lateral flap anastomosed to the anterior tibial artery (ATA) and its vena comitantes (VC) and the medial was anastomosed to the posterior tibial artery (PTA) and its vena comitantes. The donor site was closed primarily.

**Figure 2:**
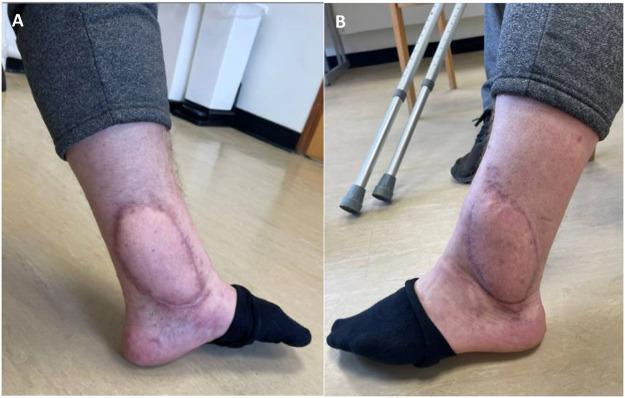
Flap healthy at nine months post-surgery. A – Medial ankle flap. B – Lateral ankle flap.

Nine-months post operatively, the patient is recovering well, and the flap is healthy (Fig. 2). There is a degree of lymphoedema which is awaiting investigation with a lymphoscintography and subsequent consideration for lymphaticovenous anastomosis. This could theoretically be attributed to venous insufficiency as a consequence of the use of two pairs of VC from the PTA and the ATA of the lower limb.

## Discussion

First described by Song in 1984, the ALT flap is considered a workhorse flap for lower limb reconstruction due to its ability to provide reliable and robust coverage for large defects^1^. While initially described as being based on the descending branch of the LCFA, Wong et al.^2^ identified the presence of an oblique branch of the LCFA that could be used as an alternate vascular pedicle.

In recent years, the concept of chimeric ALT flaps, those incorporating multiple tissue components based on individual perforators arising from a shared vascular pedicle, has broadened the versatility of this reconstructive option.^3^ In 2006, Chou described the harvest of two ALT flaps, each with their own pedicle from the descending branch of the LCFA for reconstruction of lower extremity and oral cavity defects.^4^ Huang et al.^5^ subsequently employed a similar technique for bilateral buccal mucosal reconstruction. Building on this, Ahmad et al.^6^ described the harvest of split ALTs for the purpose of breast reconstruction following excision of a Phylloides tumour, with one flap pedicle based on the descending branch of LCFA and other on the oblique branch. Benefits of split ALTs include the ability to resurface complex, irregularly shaped defects and the potential to harvest smaller skin paddles, thereby facilitating primary closure of the donor site. Moreover, in the context of oncological reconstruction, this approach preserves the contralateral ALT donor site for potential use, offering added reconstructive flexibility.^4^, ^5^, ^6^

To date, there is limited published evidence on the application of split ALTs with independent pedicles in lower limb trauma reconstruction. In this case, the patient presented with two separate soft tissue defects of the ankle, while the anterior ankle skin remained intact. Preservation of this anterior tissue precluded the use of a single chimeric flap due to concerns over pedicle compression and flap congestion. Given the patient’s lower limb vascular anatomy was preserved, we elected to proceed with two independent ALT flaps, each based on a separate branch of the LCFA. This technique allowed for effective reconstruction of both defects using a single donor site that was closed primarily.

We propose that in selected patients with anatomically distinct bilateral simultaneous medial and lateral defects, split ALT free flaps with independent pedicles provide a reliable, aesthetically favourable and donor-site sparing reconstruction solution.

## Consent

Informed written and verbal consent was obtained from the patient for publication of this case and associated images.

## Declaration of competing interest

The authors declare that they have no known competing financial interests or personal relationships that could have appeared to influence the work reported in this paper.
